# Suboxone Treatment and Recovery Trial (STAR-T): Study Protocol for a Randomised Controlled Trial of Opioid Medication Assisted Treatment with Adjunctive Medication Management Using Therapeutic Drug Monitoring and Contingency Management

**DOI:** 10.1155/2019/2491063

**Published:** 2019-03-05

**Authors:** Hesham Elarabi, Abuelgasim Elrasheed, Ahmed Ali, Mansour Shawky, Nael Hasan, Tarek A. Gawad, Abdu Adem, John Marsden

**Affiliations:** ^1^Addictions Department, Institute of Psychiatry, Psychology and Neurosciences, King's College London, 4-Windsor Walk, ASB, Denmark Hill, SE5 8BB, London, UK; ^2^National Rehabilitation Centre, UAE, P.O. Box 55001, Abu Dhabi, Shakhboot City, UAE; ^3^College of Medicine and Health Sciences, United Arab Emirates University, P.O. Box 15551, Alain, AD, UAE; ^4^Addictions Department, Institute of Psychiatry, Psychology and Neurosciences, King's College London, Addiction Sciences Building, 4 Windsor Walk, Demark Hill, London, Denmark Hill, SE5 8AF, UK

## Abstract

**Introduction:**

Opioid assisted treatment (OAT) with buprenorphine (BUP) is front-line medical maintenance intervention for illicit and prescription opioid use disorder (OUD). In many clinics, opioid medication is dispensed for several days for self-administration. This provides flexibility to the patient but may compromise the effectiveness of OAT because of nonadherence or medication diversion. OAT can be delivered as an entirely supervised intervention, but many patients discontinue treatment under this arrangement and dispensing costs may be prohibitive. An alternative is to enable patients to receive take-home doses contingent on OAT adherence guided by a medication management framework using Therapeutic Drug Monitoring (TDM) alongside negative urine drug screens (UDS) to provide evidence of abstinence. TDM is recommended to monitor adherence with BUP but it has not been applied in OAT programs and evaluation research to date.

**Methods:**

The Suboxone Treatment and Recovery Trial (STAR-T) is a single site, 16-week, parallel-group, randomised controlled trial. The aim of the study is to determine the effectiveness of a medication management framework including TDM and UDS to enable patients enrolled on outpatient OAT (with buprenorphine/naloxone [sublingual film formulation; BUP/NX-F; Suboxone™]) to receive stepped take-home doses. Following stabilisation during inpatient care, adult participants with illicit or prescription OUD were allocated (1:1) to receive (1) BUP/NX-F plus medication management for take-home doses based on TDM, UDS, and contingency management protocol (the experimental group) or (2) BUP/NX-F plus UDS only (treatment-as-usual, the control group). The primary outcome is the mean percentage of negative UDS over 16 weeks. The secondary outcome is treatment retention defined as completion of 16 weeks of OAT without interruption. There will be an exploratory analysis of the association between participant characteristics, clinical data, and outcomes.

**Conclusions:**

Providing BUP/NX-F take-home doses contingent on adherence and opioid abstinence may enable OAT to be delivered flexibly and effectively.

**Trial Registration:**

ISRCTN41645723 is retrospectively registered on 15/11/2015.

## 1. Introduction

 The annual mortality rate among the illicit opioid use population is 1%, a rate 10-fold greater than the general population [1]. The front-line, evidence-supported pharmacotherapy for opioid dependence [2] or opioid use disorder [3] (OUD herein) is oral methadone or sublingual tablet buprenorphine (BUP) maintenance [4]. On average, this opioid assisted therapy (OAT) is associated with clinically meaningful suppression of nonmedical opioid use and drug injection [5]. Studies have shown that patients who take buprenorphine/naloxone (BUP/NX) 80% of the time or more have a 10-fold increase in the odds of heroin abstinence [6] and those considered as compliant with BUP medication provide more opioid negative urine screens [7].

The effectiveness of OAT is hampered by treatment nonadherence and diversion, prescribing lower than the doses need [8], and also early discontinuation [9]. The medication dispensing policy may influence these negative outcomes. Medication can be administered either under direct supervision or flexibly, with the patient given the opportunity to receive “take home” doses for self-administration, contingent on medication adherence, and providing evidence of illicit opioid abstinence [10, 11]. A fixed policy of only dispensing medication under supervision substantially reduces the likelihood of medication diversion; but this may prove unpopular among patients and lead to drop out [9]. Medication dispensing costs may also be prohibitive for many clinical services [12]. Patients respond well to a medication management framework using flexible dosing and behavioural reinforcement (contingency management [CM] is associated with good adherence [11]), although there remains a risk of medication diversion [9]. The current evidence shows no difference between the fixed and the flexible OAT prescribing practice in reducing opioid use or enhancing retention in treatment. This evidence however was judged to be of low quality [13].

Several patient characteristics are associated with sub-optimal OAT response. Younger patients [14] and those with unstable housing [15] tend to have a higher risk of treatment discontinuation. Co-occurring mental health disorders have a prevalence of 40 to 55% in this clinical population [16] and may be associated with compromised treatment response [17]. In particular, depression and anxiety disorders are often reported to predict treatment discontinuation [18] and heroin use [18]. Personality disorders (particularly borderline personality disorder) are associated with poor prognosis of substance use treatment [19]). An impulsive personality trait has been observed to predict noncompletion of SUD treatment [20]. Sleep disorders are associated with day-time dysfunction in the heroin using population which may compromise patient engagement in treatment [21].

Buprenorphine and naloxone (BUP/NX; ratio 4:1; and a sublingual film formulation [Suboxone®; BUP/NX-F developed for rapid dissolution]) have been developed for maintenance OAT with the aim of suppressing the likelihood of illicit opioid injecting (because the opioid antagonist naloxone may cause opioid withdrawal) and maximising adherence (because BUP/NX-F is very hard to remove once placed under the tongue). In contrast to BUP mono therapy, there is evidence that these alternative formulations deliver further reduction in diversion [22]. A ‘pill or medication count' practice has also been recommended as part of the effort to increase medication adherence [23] but, to date, there are no reported randomised controlled trials.

Therapeutic Drug Monitoring (TDM) is a patient centered and precision medicine tool that involves quantitation and interpretation of medication blood concentrations with necessary dose adjustments to optimise treatment outcomes [24]. It has been applied in neuropsychiatry to enhance outcomes of antiepileptics [25], antipsychotics [26], and mood stabilisers [27] and for monitoring drug-drug interactions [28]. The potential value of TDM in OAT has been recognised as a monitor of compliance [24] yet there is no consensus or guidelines on how it should be clinically implemented and there have been no published clinical trials.

Against this background, the present study will determine the effectiveness of TDM, urine drug screens (UDS), and medication take-home dosing by CM. To our knowledge, there have been no trials that use these adjunctive elements in OAT. In this protocol paper, we describe the design, methods, procedures, and strengths and limitations for a randomised controlled trial to determine the clinical effectiveness of an adjunctive medication management protocol for OAT with BUP/NX-F.

## 2. Methods

### 2.1. Study Design, Population, and Setting

The Suboxone Treatment and Recovery Trial (STAR-T) is a single centre, 16-week outpatient intervention, two-arm, pragmatic, phase IV randomised controlled trial of OAT and adjunctive TDM for OUD. The study population is adults (≥ 18 years) with current OUD.

The study setting is the specialist OUD treatment and care programme operated by National Rehabilitation Centre (NRC), Abu Dhabi, United Arab Emirates (UAE; www.nrc.ae). In the UAE, use and combination use of heroin, morphine, and illicit tramadol are the most prevalent [29, 30]. The NRC treatment programme includes an inpatient unit for assessment and management of medical and mental health comorbidities with (poly) substance and alcohol use disorders [29–31]. In 2002, the NRC introduced OAT with BUP with induction and stabilisation procedures conducted in the inpatient unit. However, following concerns about medication diversion in 2011, the NRC suspended all new admissions to OAT pending the development and findings from the present study.

Following a standard of care protocol for OAT, all participants will first complete inpatient care (up to four weeks) to achieve medically supervised withdrawal and stabilisation on BUP/NX-F and to estimate the BUP Elimination Rate (EL.R). After study enrolment and prior to discharge, participants will be randomly allocated (1:1: using an online randomisation service [32] with no stratification) to an experimental group (that immediately received 16 weeks of outpatient BUP/NX-F maintenance, standard case management, and manualised adjunctive medication management with TDM monitoring and CM) or to a treatment-as-usual, control group (that immediately received BUP/NX-F and standard case management and usual medication management only). Using ongoing medication management, TDM, and CM protocol, participants in the experimental group will be able to receive up to four weeks of medication on a take-home basis. All participants will continue to receive ongoing treatment after 16 weeks as usual.

The trial will follow the ethical principles of the World Medical Association's Declaration of Helsinki for research involving human subjects and is registered with the ISRCTN (number: 41645723). The study will adhere to the medical research guidelines of the Department of Health of Abu Dhabi [33] and the CONSORT guideline extension for pragmatic randomised controlled trials [34]. Good clinical practice training will be provided in the UAE and in the United Kingdom by King's Health Partners Clinical Trials Office (https://www.khpcto.co.uk).

The study protocol, participant information sheet (describing the study rationale, design and procedures), participant consent form, and clinical research forms have been approved by the Institutional Review Board of the National Rehabilitation Centre, Abu Dhabi (number: NRC/2/2014; granted April 2014; first participant enrolled on 15.9.2014).

### 2.2. Study Aims

The primary aim of this pragmatic study is to determine if BUP/NX-F with adjunctive TDM is clinically superior to BUP/NX-F only in terms of reduced opioid use during outpatient treatment.

In addition to determining group differences on OAT retention, there are two exploratory secondary aims: (1) to determine if there are associations between participant demographics, two BUP parameters (elimination rate and dose), and opioid use and treatment retention; and (2) to determine if there are associations between patient psychosocial functioning and opioid use and treatment retention.

STAR-T also includes an exploratory health economic (cost-benefit and cost-effectiveness) evaluation. This component of the study will be described and reported separately.

### 2.3. Participant Eligibility and Enrolment Procedure

The participant inclusion and exclusion criteria for the study are summarised in Table 1. Screening of patients for study eligibility was carried at intake before admission to the inpatient detoxification unit.

### 2.4. Research Assessments

The following measures were recorded prior to randomisation (baseline), during the outpatient treatment phase (as shown in parentheses; see Table 2 for summary)

#### 2.4.1. Urine Drug Screen (with Confirmatory Testing; Baseline and Every Clinic Visit) and BUP Level Determination (See Section 2.7 for Frequency of Administration)

A 5-minute, point-of-care immunoassay UDS test will be used that is US FDA approved and Clinical Laboratory Improvement Amendments (CLIA) waived for the following drugs screen in urine: opioids (morphine for illicit heroin), propoxyphene, tramadol, oxycodone, benzodiazepines, tricyclic antidepressants, psychostimulants (d-amphetamine, methyl-amphetamine, MDMA, cocaine), cannabinoids, phencyclidine, and BUP. All urine samples were collected under supervision, and positive screens were sent for confirmatory analysis at the laboratory using Gas Chromatography Tandem Mass Spectrometry. BUP levels were detected and quantified by Liquid Chromatography Tandem Mass Spectrometry (Schimadzu Scientific Instruments) with a Raptor C18 analytical column (Restek Corporation; 9304A12).

#### 2.4.2. Clinical Opioid Withdrawal Scale (COWS [35]; See Table 2)

The COWS is an 11-item clinician-administered scale which assesses opioid withdrawal signs and symptoms (a higher score indicates more severe opioid withdrawal).

#### 2.4.3. Pupil Reflexes (PLA Inc. 2000; Neuroptics, https://neuroptics.com; See Table 2)

A hand-held camera captures three pupil reflexes [36]: (1) maximum pupil diameter reading before exposure to light (before contraction). (2) minimum pupil diameter reading after exposure to light (after contraction), and (3) maximum and average constriction velocity, dilation velocity, and time to 75% recovery of pupil diameter.

#### 2.4.4. Patient Health Questionnaire (PHQ-9 [37]; See Table 2 for Frequency of Administration)

The PHQ-9 is as a validated, self-administered 9-item scale recording frequency of depression-related symptoms according to the DSM-IV depression criteria using responses over the past two weeks. The PHQ-9 screens for mild, moderate, moderately severe, and severe depression at cut-offs of 5, 10, 15, and 20, respectively. A validated Arabic version downloaded from the PHQ Screeners webpage [www.phqscreeners.com] was used in the present study.

#### 2.4.5. Generalised Anxiety Disorder (GAD-7 [38]; See Table 2 for Frequency of Administration)

The GAD-7 is a validated, self-administered 7-item scale recording frequency of anxiety-related symptoms according to the DSM-IV anxiety criteria using responses over the past two weeks. The GAD-7 screens for mild, moderate, and severe anxiety at cut-offs of 5, 10, and 15, respectively. A validated Arabic version downloaded from the PHQ Screeners webpage [www.phqscreeners.com] was used in the present study.

#### 2.4.6. Barratt Impulsiveness Scale (BIS-11 [39]; See Table 2 for Frequency of Administration)

The BIS-11 is a validated, 30-item self-administered questionnaire that assesses three impulsiveness subtraits: nonplanning, motor, and attention. Items are rated over a four-point scale (“never” to always; scored 0 to 4 total score range: 0 to 120). A higher score indicates higher trait impulsiveness.

#### 2.4.7. Personality Disorder Screener (PDS [40]; See Table 2 for Frequency of Administration)

The PDS is a validated, clinician-administered 34-item “true”, “false”, or “do not know” checklist. Scoring follows the ICD-10 criteria to screen for three clusters of personality disorders: Cluster A (Odd or Eccentric), Cluster B (Borderline Personality), and Cluster C (Anxious Personality).

#### 2.4.8. Addiction Severity Index (ASI-Lite Version [41]; See Table 2 for Frequency of Administration)

The ASI-lite is a validated, semistructured interviewer administered outcome evaluation instrument that assesses seven addiction severity domains over the past 30 days (medical and employment and social status; alcohol use; drug use; family; legal; and mental health). The tool generates a composite score for each domain (ranging from “0 to 1”), with higher scores indicating higher problem severity.

#### 2.4.9. Work and Social Adjustment Scale (WSAS [42]; See Table 2 for Frequency of Administration) 

The WSAS is a validated, 5-item self-reported scale that measures perceived personal, social, and occupational impairment caused by a clinical problem (OUD in the present study). Each item is rated using an 8-point scale (“0” [no impairment] to “8” [full impairment]; total score range: 0 to 40). A score of “10 to 20” indicates significant impairment and a score of “21 to 40” reflects severe impairment.

#### 2.4.10. Pittsburgh Sleep Quality Index (PSQI [43]; See Table 2 for Frequency of Administration)

The PSQI is a validated, self-administered tool that evaluates sleep quality across seven categories with items rated on a 3-point scale (total score 0 to 27). A higher score reflects worse sleep quality and the cut-off score for sleep disorders is “5”. The published Arabic version by Suleiman and colleagues in 2010 [44] will be utilised.

#### 2.4.11. Minnesota Cocaine Craving Scale (MCCS [45]; See Table 2 for Frequency of Administration)

The MCCS is a validated, 5-item scale measuring the following aspects craving: intensity, duration, frequency, change from last week/day, and how the medication has helped. The MCCS was adapted to record “opioids” (MOCS) for the present study.

### 2.5. Patient Education and Medication Management Materials

The following materials were developed to support trial implementation and fidelity: 

*(1) Medication Education Leaflet*. An educational material on how to use BUP/NX-F was developed for patient medication education [46] and following the BUP clinical practice published by the US Department of Health and Human Services-Substance Abuse and Mental Health Services Administration [37]; 

*(2) Emergency Card*. As a safety measure, a wallet-size hard card was developed for health care professionals who attend participants in the state of emergency specifying the prescription of BUP/NX-F; 

*(3) Patient Diary/“Recovery Passport”*. A passport-sized diary was developed based on the patient health engagement model [47], self-management, and principles of CM to include material on self-assessment, a log of BUP/NX-F dosing (validated by the participant and a family member), and a log of clinic visits for UDS and results; 

*(4) Patient Counselling Checklist*. A 19-item checklist is developed to guide medication counselling [48]; and 

*(5) Medication Management Manual*. A manual was developed to structure the medication management sessions for the experimental group. This was based on material developed for US trials of alcohol [49] and opioid pharmacotherapy [50] and included monitoring forms to individualise interventions and text to guide the clinician's interactions with the patient.

### 2.6. Procedures

#### 2.6.1. Buprenorphine/Naloxone Induction and Stabilisation

On the first day of admission to the inpatient unit, participants' pupil reflexes will be measured to provide a baseline for monitoring craving and medication response. Then, at the first sign of withdrawal, a three-day or five-day supervised BUP/NX-F induction will commence for individuals with morphine/heroin use disorder or pharmaceutical OUD, respectively. The longer induction period for the latter group reflects the relatively longer half-life of these products compared to heroin.

BUP/NX-F will be initiated at a dose of 2 to 4 mg will be used for those with a COWS score of <10. The participant will be closely observed during the first 4 hours to signs of precipitated withdrawal, together with regular pupil reflex monitoring and COWS assessments. The participant's dose will be increased by 2 to 4 mg to a maximum of 8 mg in the first 24 hours. On the second day of induction, the dose will be increased by 4 to 6 mg every 4 to 6 hours to a maximum of 24 mg per day.

With an achieved COWS score of <5, the participant will be transferred to an “early recovery unit” a step-down phase to achieve BUP/NX-F stabilisation. Each participant will be assigned to a personalised dosing schedule (i.e., daily, alternate-day [Alt-D] or thrice-weekly [TIW]) followed by further dose adjustment as required. Those who continue to report distressing opioid craving or do not tolerate their dosing schedule satisfactorily will be transferred to another (usually more frequent) schedule. The published correlation of patient characteristics and BUP/BUP/NX maintenance doses [51, 52] will serve as the theoretical basis for this regimen and the daily, or the total 24-hour dose, required to achieve a COWS scores <5 will be used as the reference for estimating non-daily doses.

**Figure 1:**
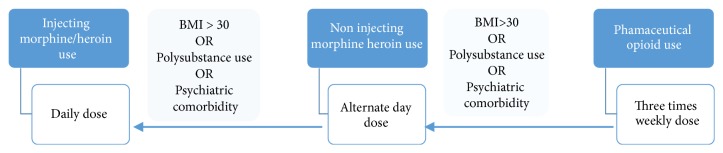
Dose assignment and stabilisation.

The Alt-D schedule will be a 3 × 48-hour dose and 1 × 24-hour dose. The TIW schedule will be a 2 × 48-hour dose and 1 × 72-hour dose (calculated as 3 × 24-hour dose at a maximum of 32 mg) [53, 54]. Clinical determination of the dosing schedule was informed by illicit injecting status (i.e., participants injected street heroin/morphine will receive daily doses and noninjected heroin and morphine users received alternate daily doses) while prescription opioid users (nonpolysubstance users) will receive TIW doses. Additionally, participants with severe psychiatric comorbidity, polysubstance use, or a body mass index (BMI) of ≥30 will be placed on the next higher frequency schedule (e.g., from TIW to ALT-D). Fine dose adjustments will be guided by self-report participant comfort, sleeping, craving, and pupil reflexes. Participants continuing to observe craving and or signs of withdrawal will be transferred to the next frequent dosing schedule towards the daily schedule as illustrated by Figure 1.

#### 2.6.2. Estimating the Buprenorphine Elimination Rate Constant

Maintenance of a BUP/NX-F dose without change for two weeks will be taken to indicate that a BUP steady state concentration (SSC) [55] has been achieved for participants receiving a daily or Alt-D dosing schedule. A longer period of 21 days (or an equivalent of 9 doses) will be needed to achieve SSC for participants assigned to TIW. Then, applying the function for first order kinetics (Figure S1), the BUP EL.R will be estimated by

(1) examination of the peak BUP plasma concentration measured 40 minutes after the dose on day 19 and day 22 [peak concentration, C-max]; and

(2) examination of BUP trough levels measured 30 minutes prior administering BUP/NX-F on day 21 and day 24 (trough concentrations, C-min). The replication of two trough concentrations will be taken to confirm that a SSC has been achieved. It is possible that additional samples will need to be drawn until SSC is confirmed. The reliability of the first order pharmacokinetic (PK) model to accurately predict BUP levels will be evaluated in the first 15 participants recruited to the study in the form of an internal pilot study for TDM. After confirming the reliability of the PK model in all 15 participants, the study will proceed to definite recruitment and results from the pilot will be included in the study analysis. Details of the laboratory assay and clinical procedures for this step will be presented separately.

#### 2.6.3. Interventions for Experimental Group

In week one of outpatient care, participants allocated to the BUP/NX-F and TDM (experimental) group will receive directly supervised doses according to their dosing schedule. Participants are required to randomly provide a minimum of three negative UDS during these visits on a random basis. If the participant is able to meet this this requirement, a CM protocol will enable them to receive a one-week prescription of medication for self-administration at home. On return for their prescription to be refilled, a negative UDS will enable the participant to receive a two-week prescription and then a three-week prescription. On the other hand, if the participant is not able to meet the initial requirement (i.e., they fail to attend all appointments or provide at least three negative UDS) they will receive supervised dosing for another week (five days for daily dosing schedule and two days of take home for the weekend). At any point, a positive UDS will either hold the participants on five-day supervised dosing or step them down to this arrangement from the two-week or three-week dosing arrangement.

For the TDM element, after the participant has been able to earn a two-week take-home prescription, a blood sample will be collected during a clinic visit for laboratory to measurement of BUP level labelled the “observed concentration”. On the sample collection day, participants will be strictly advised not to take their BUP/NX-F dose and the quantity of medication dispensed will be accounted for by the pharmacy. The exact time of blood sample collection, the time of the last BUP/NX-F dose taken/administered, and the established BUP EL.R will be applied in the function of first order kinetics (Figure S1) to predict the participant's concentration labelled as “predicted concentration” of BUP. If the observed and predicted concentration values do not differ by more than 15%, the participant will be classified “adherent”. Participants who are adherent will be stepped up to a three-week take-home prescription. All nonadherent participants will be stepped down to a one-week take-home prescription.

On a random basis, all participants who attain the three-week take-home prescription will be invited to visit the clinic between scheduled visits to give a blood sample for BUP testing and also to take a UDS. Nonadherent or nonabstinent participants will be stepped down to two-week take-home prescription while adherent and abstinent participants will receive a four-week take-home prescription at the next scheduled visit (the maximum permitted for the trial). During outpatient treatment, participants who are assessed as both nonadherent and nonabstinent will be reset to supervised dosing.

During the first week of outpatient care, two medication management foundation sessions will be scheduled to help participants (1) understand the importance of taking BUP/NX-F as prescribed, (2) become aware of BUP/NX-F mechanism of action and ways to monitor withdrawal signs and adverse events, (3) recognise and cope with cravings, and (4) build and sustain motivation for abstinence. Then, medication management maintenance sessions will be offered in response to the following four participant conditions: 

*Abstinent and Adherent*. Discussion is held to reinforce and motivate continued abstinence and medication adherence. Prescription for take-home doses will be extended step-wise to a maximum of four weeks. 

*Abstinent but Nonadherent*. Discussion is held to reinforce and motivate continued abstinence, remind the participants about the value of adherence, identify the source of nonadherence and strategies to improve adherence. A follow-up call on the agreed tasks will be arranged and prescription for take-home doses will be stepped down by one level. 

*Nonabstinent but Adherent*. Discussion is held to reinforce and motivate medication adherence and remind participants about the value of abstinence. The context and triggers for using will be assessed and relapse prevention strategies discussed. Additionally, comorbid conditions and social situations will be evaluated with referral to ancillary services. The prescription for take-home doses will be stepped down by one level. 

*Nonabstinent and Nonadherent*. Functional assessment of relapse/lapse will be performed, with referral to an appropriate service if co-occurring conditions were identified. Engagement of family members and close network will be sought to encourage retention. The participant will be transferred to five-day supervised BUP/NX-F dosing.

#### 2.6.4. Interventions for the Control Group

Participants randomised to the TAU control group will receive BUP/NX-F dosing according to preference of daily, twice weekly, or every two weeks alternatives without a mandatory supervised dosing period. In this group, take-home doses will be provided contingent on provision of negative UDS only without following a fixed protocol. Medication management sessions will not be delivered according to structured manual and no monitoring of BUP levels will be done.  Table 3 contrasts the interventions under the experimental and control group.

### 2.7. Study Outcomes

#### 2.7.1. Primary Outcome

The primary outcome measure for the study will be the count of negative opioid UDS (excluding BUP) over 16 weeks. Scheduled appointments for a UDS that are missed will be conservatively imputed as positive for opioids.

#### 2.7.2. Secondary Outcome

Retention as defined as completion of the 16-week outpatient treatment without interruption. All participants who miss three consecutive appointments will be judged to have discontinued treatment.

#### 2.7.3. Exploratory Outcomes

There is change in psychosocial functioning from baseline at the end of the 16-week treatment. There is correlation of participant demographics, clinical data, BUP EL.R, and BUP/NX-F maintenance dose with the primary and second outcome.

### 2.8. Sample Size Calculation

Given the novelty of the study, the sample size was estimated indirectly with reference to the relevant CM literature as reviewed by the UK National Institute of Care Excellence (NICE Clinical Guideline 51; Appendix 15 [56]. The study was powered for 80% to detect a difference of 3 weeks opioid abstinence as evaluated by NICE for three trials (an odds ratio in favour of CM of 2.56; 95% CI 1.76 to 3.72). Using this pooled effect, uplifted by 15% for attrition and with a 5% two-sided alpha, it was estimated that 92 participants should be allocated to the experimental and control group.

### 2.9. Statistical Analysis of the Primary and Secondary Outcomes

Data for the whole population and by study group will be analysed for mean, standard deviation, 95% confidence interval, and range. With no interim analysis, all statistical analyses will be pragmatically done according to intention-to-treat principle. All analyses will be conducted using two-sided 5% significance test. A fixed-effects logistic model will be applied to analyse the primary outcome. To analyse between group differences, student* t*-test will be conducted for normally distributed data, or a Mann-Whitney test. For the primary outcome, a planned sensitivity analysis will determine the impact of the imputed or complete case [57]. Alternatively, reported observed opioid screens or actual screens without imputation will be analysed between group differences. Secondary outcome will be evaluated using proportional hazards regression model and Chi-Square test will be performed to measure between group differences.

### 2.10. Analyses for Exploratory Outcome

Bivariate correlation tests to explore correlations with study outcomes will be performed. Specifically, Pearson's correlation will be applied for continuous and Spearman's rho test for categorical data. Factors demonstrating significant correlation and those with potential impact on the primary outcome will be studied for predictive power using a simple linear regression model. Finally, analysing for the effect of confounders will be done using univariate analyses for factors generating higher correlation (>0.5). The mean change from baseline scales or within group change will be analysed using a generalised linear model framework. A paired* t*-test will be conducted where normal distribution is assumed or alternatively Kruskal-Wallis test. The magnitude of change from baseline will be estimated using bias corrected Hedge's* g* effect size. Mean difference between groups will be analysed using student* t*-test where normal distribution is assumed or a Mann-Whitney test.

## 3. Treatment Monitoring

The study will be overseen by a Management and Safety Committee (MSC) and a Trial Management Group (TMG). The MSC is an independent committee and will meet quarterly to monitor participant recruitment, safety aspects, and implementation process. Reporting to the MSC, the TMG will meet fortnightly and will focus on day-to-day management of the study. An adverse event form will record the type, severity, start and end dates of each event, likely association to BUP/NX-F, and actions taken and outcome. Response to adverse events will follow the study protocol (Table S.1.). Implementation of the study procedures, data collection and management, and functions of the governance committees will be audited every 6 months. A random 5% audit of the material recorded during the medication management sessions will also be conducted with additional training provided as required.

## 4. Recruitment and Study Status

STAR-T is an ongoing study that commenced recruiting participants on 15^th^ of September 2014 and data analyses is still in progress.

## 5. Discussion

This pragmatic study will provide empirical data on the outcomes of personalising OAT in reducing opioid use and enhancing treatment retention. Personalised care was assumed by adjusting BUP/NX-F “take-home” prescriptions according to medication adherence, judged by TDM data, and drug use judged by UDS. This approach was hypothesised to optimise adherence and minimise diversion. This study evaluates the integration of TDM to provide stepped BUP/NX-F “take-home” against usual treatment. Findings from this study would contribute to the expanding OAT currently limited by poor compliance, concerns over diversion, and high cost supervised treatment [12] associated with high treatment dropout rates [6].

A practical alternative to prospectively assigning participants to BUP/NX-F dose schedules would be random assignment and stabilisation of participants followed by analysis of participant characteristics associated with each dose schedule. On the other hand, contrasting the level of medication adherence concluded by TDM with that generated by UDS would have strengthened the justification of using TDM considering the cost of both methods.

Although EQ-5D [58] is the approved tool for health utility calculations by the National Institute for Clinical Excellence (NICE) in the UK, EQ-5D does not offer the required sensitivity to assess mental health disorders of nonacute presentations. This has encouraged the authors to explore WSAS as a brief and self-administered measure of disability and inversely utility to estimate changes in quality of life (QOL). For future research we suggest the validation of WSAS against a standard tool measure of QOL like Short Form Health Survey-Arabic version (SF-36, [59])

Blood is the biological matrix identified by the TDM consensus guidelines for quantitation of BUP [24]. In blood, BUP demonstrates linear kinetics and time to peak concentration has been established [24, 60]. In contrast, detection of BUP in urine is performed but quantitation was not recommended due to erratic clearance with approximately 30% of BUP excreted in urine. Equally important, time to achieve BUP peak concentration was not established in urine unlike blood [60].

The identified strengths allowing for generalisability of results include expanded inclusion criteria and exclusion of factors reported to minimise retention, e.g., unstable housing arrangements [15]. The 16-week period could be optimal for evaluating relapse prevention. On the other hand, the extended turn-around time to report BUP levels might influence the effectiveness of CM based interventions shown to be most effective when provided within 24 hours of the behaviour [11]. Other possible limitations include applying nonstratified randomisation due to the extended stratification factors and blocks include city of residence; type and pattern of opioid use; polysubstance use; and comorbid anxiety, depression, and impulsiveness. Categorical reporting of retention limits assessing the potential value of partial completion. In the absence of consensus on defining treatment retention, we chose the most stringent definition of retention which is maintaining access to treatment at different treatment points including the end of the study [61].

## Figures and Tables

**Table 1:** Participant inclusion and exclusion criteria.

*Inclusion criteria*
For a participant to be enrolled into the study he must fulfil all the following inclusion criteria:
(1) Aged 18 and above with no upper limit (usually 64 years);
(2) Current diagnosis of OUD;
(3) Voluntarily seeking OAT treatment;
(3) Resident in the UAE;
(4) Evidence of stable accommodation.

*Exclusion criteria*
Otherwise eligible patients will be excluded from the study for any of the following:
(1) Benzodiazepine use in excess of 20 mg daily diazepam equivalent in the past 28 days;
(2) Known naloxone or BUP hypersensitivity;
(3) Pregnancy;
(4) Hepatic impairment (elevation of liver function tests three times normal);
(5) Suicide attempt in past 12 months;
(6) Involvement in criminal justice system which is likely to result in arrest and incarceration;
(7) Uncontrolled severe mental or physical illness judged to compromise safety;
(8) Mini Mental State Examination score < 17 indicating cognitive dysfunction.

**Table 2:** Schedule for administering study measures.

Tool/ Screen	Baseline Intake	Inpatient		16 week outpatient study period				
		Detoxification (Daily)	Stabilisation (Weekly)	Week 1 to 4	Week 5 to 8	Week 9 to 12	Week 13 to 16	Week 16 End of study
Eligibility Screen	x							

MCCS	x	x	x	x	x			

Pupil Reflexes	x	x	x	x	x		x	x

COWS	x	x	x	x				

PHQ-9	x			x		x		x

GAD-7	x			x		x		x

BIS-11	x			x				x

PSQI	x		x	x	x	x	x	x

WSAS	x			x				x

PDS	x			x				x

ASI-Lite	x			x				x

MCCS: Minnesota Cocaine Craving (adapted for opioids); PHQ-9: Patient Health Questionnaire; COWS: Clinical Opioid Withdrawal Scale; GAD-7: Generalised Anxiety Disorder; BIS-11: Barrett Impulsiveness Scale; WSAS: Work and Social Adjustability Scale; PDS: Personality Disorder Screen; ASI-Lite: Addiction Severity Index-Lite.

**Table 3:** Interventions under study groups (TDM; TAU).

Intervention	Randomisation Group	
	TDM	TAU
Induction	Yes	Yes

Stabilisation	Yes	Yes

Baseline assessments	Yes	Yes

Estimating BUP Elimination Rate	Yes	Yes

Medication education	Yes	Yes

Emergency card	Yes	Yes

*Outpatient DOT*	*Yes*	*No*

*Outpatient medication management manualised intervention*	*Yes*	*No*

UDS at outpatient care	Yes	Yes

Providing prescription take-home doses	Contingent on UDS & TDM (i.e., abstinence & adherence)	Contingent on UDS only

*Stepped BUP/NX-F take-home doses*	*Yes*	*No*

Maximum take BUP/NX-F home doses	*4 weeks*	*2 weeks*

*TDM *	*Yes*	*No*

Periodic study assessments	Yes	Yes

End of study assessments	Yes	Yes

Psychosocial support	Yes	Yes

BUP: buprenorphine; DOT: directly observed treatment; UDS: urinary drug screen; TDM: therapeutic drug monitoring.

## Data Availability

Data supporting the findings of this study are available from the NRC (www.nrc.ae) but restrictions apply to the availability of data used under license for the current study. Public availability of materials is not applicable due to concerns of violating patient confidentiality. However, data are available from the corresponding author upon reasonable request and permission of the NRC.

## Additional Points

*Changes Made to the Protocol*. The EQ-5D was excluded from the list of tools implemented in the study and was reported to the IRB in December 2014.

*Safety Reporting, Participant Withdrawal, and Treatment Stopping Rules*. All adverse reactions are recorded. Nonfatal or life threating events are reported within 15 days of discovering the reaction. Fatal or life-threatening events are immediately reported with additional information reported within eight days. Adverse events managed according to the provisions of Table S1 are assessed for level of seriousness and likely association with study interventions. Participants can withdraw from treatment at any time, and the reason for withdrawal is recorded. Participants may also withdraw from the study for safety reasons (e.g., in the event of serious adverse events and reactions, or medical conditions which require acute or intensive hospital procedures). Finally, a decision to stop the trial prematurely might be taken further to emerging data.

*Safety Reporting, Participant Withdrawal and Treatment Stopping Rules*. All adverse reactions are recorded. Non-fatal or life threating events are reported within 15 days of discovering the reaction. Fatal or life-threatening events are immediately reported with additional information reported within eight days. Adverse events managed according to the provisions of Table S1, are assessed for level of seriousness and likely association with study interventions. Participants can withdraw from treatment at any time, and the reason for withdrawal is recorded. Participants may also be withdrawn from the study for safety reasons (e.g. in the event of serious adverse events and reactions, or medical conditions which require acute or intensive hospital procedures). Finally, a decision to stop the trial prematurely might be, taken further to emerging data.

## Supplementary Materials

Supplementary Materials Table S1. Management of adverse events. Figure S1. Elimination rate equation.
