# Supplementary material for: Suboxone Treatment and Recovery Trial (STAR-T): Study Protocol for a Randomised Controlled Trial of Opioid Medication Assisted Treatment with Adjunctive Medication Management Using Therapeutic Drug Monitoring and Contingency Management
Source: J Addict. 2019 Mar 5;2019:2491063. doi: 10.1155/2019/2491063 (PMC6425325; doi:10.1155/2019/2491063)
Supplement: Supplementary Materials — Table S1. Management of adverse events. Figure S1. Elimination rate equation. [file 2491063.f1.pdf]

Supplementary tables and figures:

Table S1. Management of adverse events

Figure S1. Elimination rate equation

Table S1. Adverse Event Management

**Event**	**Management**
**Headache**	Paracetamol 1g qid (prn)
**Pain**	Paracetamol 1g qid (prn)
**Asthenia**	Evaluate sleep and general life style. Counsel the patient on health promotion
**Constipation**	Bisacodyl 5mg prn
**Insomnia**	Evaluate the cause of insomnia. Check if new drugs were added or dose adjustment to current concurrent medications Evaluate sleep wake cycle. Check for withdrawal syndrome if present increase the dose by 4 mg. If absent prescribe hyrdoxizine 10 mg and if no response is observed in 3 days switch to Zopiclone 7.5 mg prn.
**Orthostatic Hypotension**	Discuss possible reasons with his primary physician. Advise the patient not to abruptly stand from supine or sitting positions
**Dizziness**	Discuss possible reasons and interventions with primary physician.
**Abdominal Pain**	Hyoscine HBr TID
• **Nausea Vomiting**<br>• **Dyspepsia**	• Assess severity. Advice on food intake. Domperidone 30 mg (Peripheral Dopamine Blocker)<br>• Esmoperazole 20 mg prn

Figure S1. Eliminate rate equation and derivation

$$Cpss = Co.e^{-kt}$$

Where,

- C-o is the peak plasma concentration of BUP
- Cpss is the trough concentration measured at steady state or at any point in time later (Ct)
- k is the elimination rate constant
- t is time in hours between collecting peak and trough concentrations

Solving for k 'elimination rate',

Solving for k:

$$\ln [Cpss] = \ln [Co].- k*t$$

$$\ln [Cpss] = -kt + \ln [Co]$$

$$-kt = \ln [Cpss / Co]$$

Therefore,

$$-k = \ln [Cpss / Co]/ t$$
